# Quality divergence of Yunnan upland rice versus Jiangxi and Heilongjiang paddy rice revealed by physicochemical, sensory, volatile and metabolomic profiling

**DOI:** 10.1016/j.fochx.2026.104539

**Published:** 2026-09-29

**Authors:** Ying Xiao, Zhenxing Wang, Nandong Yue, Yunfei Tuo, Jiancheng Wen, Liwei Guo, Chunfang Zhao, Xiahong He, Jinggui Nie

**Affiliations:** a Yunnan Provincial Key Laboratory for Conservation and Utilization of In-forest Resource, Southwest Forestry University, Kunming 650224, China; b Forest Resources Exploitation and Utilization Engineering Research Center for Grand Health of Yunnan Provincial Universities, Kunming 650224, China; c College of Biological Science and Food Engineering, Southwest Forestry University, Kunming 650224, China; d College of Soil and Water Conservation, Southwest Forestry University, Kunming, 650224, China; e Rice Research Institute, Yunnan Agricultural University, Kunming 650201, China; f Institute of food crops, Jiangsu Academy of Agricultural Sciences, Nanjing 210014, China

**Keywords:** Plateau upland rice, Quality difference, Key odorants, Metabolomic profiles, Metabolic pathways

## Abstract

This study aimed to evaluate quality differences between Yunnan upland rice (YNUR) collected in Honghe, Pu'er, Nujiang and Qujing and paddy rice from Jiangxi and Heilongjiang by physicochemical indexes, taste analyzer, sensory evaluation, e-nose, and untargeted metabolomics. Compared with paddy rice, YNUR exhibited significantly higher amylose content (11.78–14.11%), lower expected glycemic index (60.55–69.13), and higher taste value (8.75–9.01), along with a higher retrogradation value and lower breakdown value. Its texture was characterized by high hardness, high elasticity and low viscosity. Furthermore, YNUR had higher aldehyde and ketone contents, presenting an aroma profile dominated by fruity and faint pungent notes. (Z)-2-nonenal, nonanal and 2-ethyl-3,5-dimethylpyrazine were the key differential odor compounds. Metabolomic analysis identified 573 differential metabolites and revealed significant enrichment of glycerophospholipid and amino acid metabolic pathways in YNUR. This study characterizes the quality attributes of upland rice and provides insights for the cultivation and application of water-saving rice.

## Highlights

• Metabolomics revealed the quality characteristics of Yunnan upland rice (YNUR).
• YNUR had high amylose content, low eGI, high elasticity and low viscosity.
• YNUR exhibited an intense flavor and relatively firm texture in sensory profiles.
• (Z)-2-nonenal, nonanal, and 2-ethyl-3,5-dimethylpyrazine were key odor compounds.
• ⁠⁠⁠⁠⁠⁠⁠⁠⁠⁠⁠⁠⁠⁠⁠⁠⁠⁠⁠⁠⁠⁠⁠⁠⁠⁠⁠⁠⁠⁠⁠⁠⁠⁠⁠⁠⁠⁠⁠⁠⁠⁠⁠⁠⁠⁠⁠⁠⁠⁠⁠⁠⁠⁠⁠⁠⁠⁠⁠⁠⁠⁠⁠⁠⁠⁠⁠⁠⁠⁠⁠⁠⁠⁠⁠⁠⁠⁠⁠⁠⁠⁠⁠⁠⁠⁠⁠⁠⁠⁠⁠⁠⁠⁠⁠⁠⁠⁠⁠⁠⁠⁠⁠⁠⁠⁠⁠⁠⁠⁠⁠⁠⁠⁠⁠⁠⁠⁠⁠⁠⁠⁠⁠⁠⁠⁠⁠⁠⁠⁠⁠⁠⁠⁠⁠⁠⁠⁠⁠⁠⁠⁠⁠⁠⁠⁠⁠⁠⁠⁠⁠⁠⁠⁠⁠⁠⁠⁠⁠⁠⁠⁠⁠⁠⁠⁠⁠⁠⁠⁠⁠⁠⁠⁠⁠⁠⁠⁠⁠⁠⁠⁠⁠⁠⁠⁠⁠⁠⁠⁠⁠⁠⁠⁠⁠⁠⁠⁠⁠⁠⁠⁠⁠⁠⁠⁠⁠⁠⁠⁠⁠⁠⁠⁠⁠⁠⁠⁠⁠⁠⁠⁠⁠⁠⁠⁠⁠⁠⁠⁠⁠⁠⁠⁠⁠⁠⁠⁠⁠⁠⁠⁠⁠⁠⁠⁠⁠⁠⁠⁠⁠⁠⁠⁠ YNUR were enriched in glycerophospholipid and amino acid pathways.

## Graphical abstract

**Figure:**
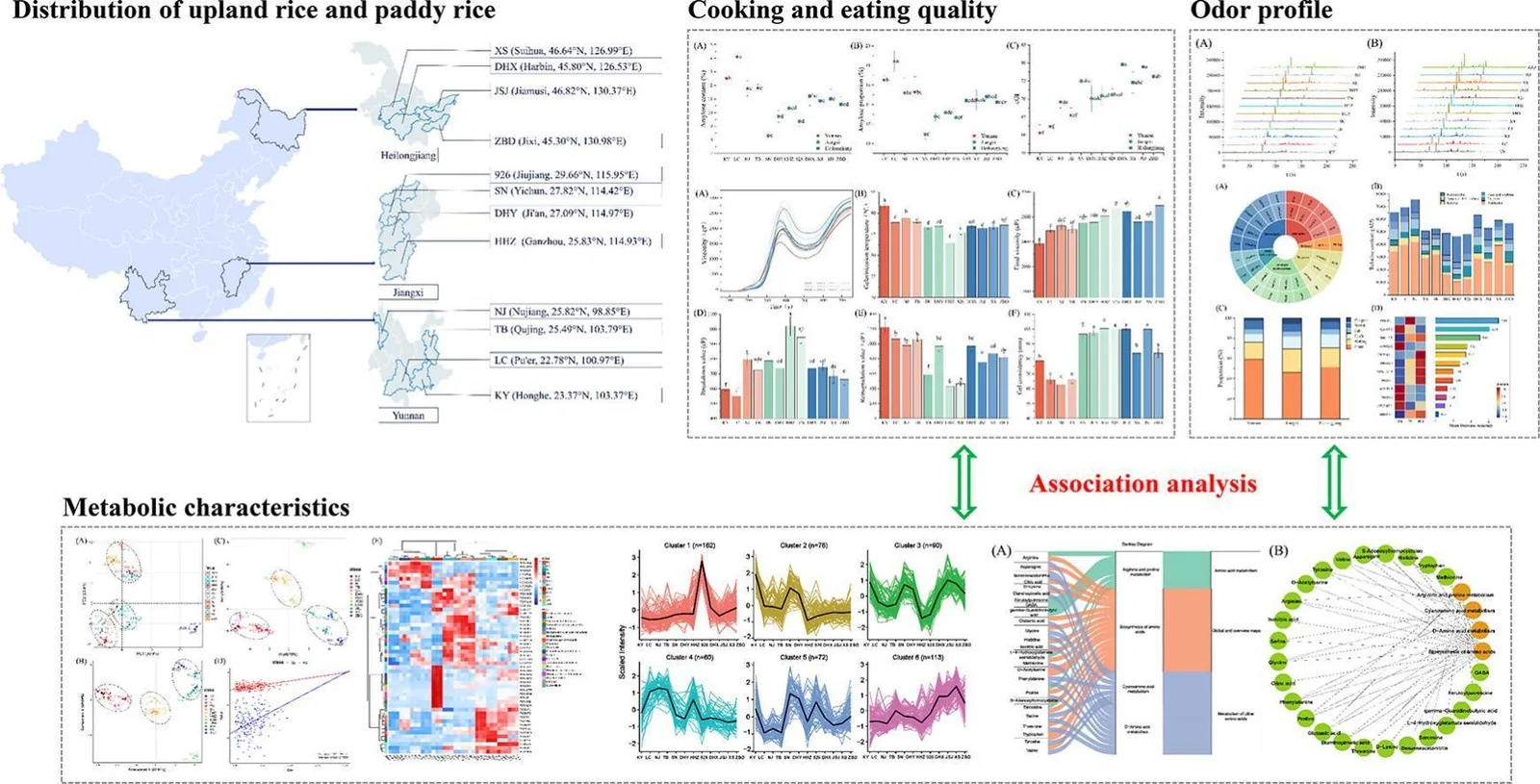

## 1 Introduction

Rice, one of the most important food crops worldwide, serves as the staple food for over 3 billion people and occupies an irreplaceable core position in the global agricultural economy and food security system (Yamamoto et al., 2024). China is the world's largest producer and consumer of rice, with a vast territory and diverse climate types (Cheng et al., 2023). In addition to its main component starch, rice is rich in various nutrients, such as protein, dietary fiber, and minerals. It also contains active ingredients, including polyphenols, anthocyanins, and flavonoids, which can effectively alleviate oxidative stress and have potential health benefits, such as antioxidant, anti-aging, and hypoglycemic effects (Yi et al., 2024).

In China, agricultural water use accounts for nearly 70% of total water consumption, among which water consumption for conventional flooded rice cultivation accounts for approximately 70% of the total agricultural water consumption, which not only leads to a serious waste of freshwater resources but also poses a threat to the agricultural ecological environment (Huang et al., 2026). The traditional flooded rice cultivation mode faces severe challenges due to seasonal droughts induced by climate change and competition for water resources between industries and agriculture (Wang et al., 2023). Upland rice features strong drought-resistance and wide adaptability, and can be cultivated on diverse land types such as sandy wastelands and saline-alkali soils (Zhang et al., 2020). The unique ecological environment of plateau regions in Yunnan, characterized by high altitude, intense solar radiation, low atmospheric pressure and seasonal drought, has shaped an agricultural pattern dominated by upland cultivation (Li et al., 2025). Conventional paddy rice cultivation is difficult to popularize in plateau areas, while upland rice serves as a characteristic food crop in this region. Therefore, in-depth research on the quality of Yunnan upland rice (YNUR) is of enormous significance for exploring water-saving rice germplasm resources and improving the efficiency of water resource utilization.

At present, research on the quality of upland rice has mainly focused on regions such as Thailand, India, and China, while the overall number of relevant studies remains relatively limited. Existing studies have mostly focused on the effects of upland and paddy cultivation conditions on the processing quality, appearance characteristics, and physiological traits of rice or on the characterization of nutritional components and gelatinization properties of upland rice in specific regions (e.g., mountainous areas) (Ngo et al., 2024; Wang et al., 2023). Additionally, some scholars have explored the effects of cultivation patterns and processing treatments on the cooking quality, fermentation characteristics, and antioxidant activities of upland rice (Wang et al., 2025). However, most existing studies have focused on upland rice from single producing areas, and its metabolic characteristics have remained unknown. Moreover, there is a lack of systematic research on the quality of plateau upland rice, especially comprehensive comparative analysis between plateau upland rice and conventional paddy rice.

This study focused on the same upland rice cultivar grown across four regions (Honghe, Pu'er, Nujiang and Qujing) in Yunnan, with eight distinct paddy rice varieties collected from Jiangxi and Heilongjiang as controls. A multi-index analytical approach was adopted to systematically compare their quality differences. We hypothesize that upland rice produced under highland upland cultivation may exhibit a lower expected glycemic index (eGI) and a characteristic profile of flavor-related metabolites. Therefore, this study mainly determined the cooking quality (amylopectin content, eGI, and gelatinization parameters) and sensory characteristics (taste analysis, sensory evaluation, and flavor profiles) of rice samples. Untargeted metabolomics analysis was further applied to detect differential metabolites. Metabolomics can provide a complementary and holistic approach for characterising food quality when integrated with conventional compositional, technological, and sensory measurements (Liang et al., 2024). This study aimed to clarify the quality characteristics of plateau upland rice in Yunnan by combining quality traits and molecular profiles, thereby providing a theoretical basis for research on the quality of plateau upland rice from different perspectives.

## 2 Materials and methods

### 2.1 Materials and chemical reagents

Twelve groups of rice samples used in this study were collected from Yunnan, Jiangxi and Heilongjiang Provinces. Their cultivars, sampling sites and agronomic management practices are shown in Table S1. The Yunnan samples consisted of the same cultivar planted across four distinct geographical regions, while samples from Jiangxi and Heilongjiang were each selected from four major cultivated varieties. A field biological replicate sampling design was adopted in this study. Each rice variety contained three independent biological replicates: sowing, cultivation management and harvesting at maturity were carried out in three separate field plots to acquire three batches of rice samples with independent origins and distinct field conditions. All samples were collected separately at the same maturity stage in 2025. The rice grains were subjected to drying (moisture content <13.5%) and preservation after removal of the husk. The appearance and processing quality were determined using whole rice, and the remaining indexes were milled into powder (100 mesh) and placed in the refrigerator at −80 °C for preservation. Straight-chain starch, α-amylase, pepsin, trypsin, and glucose kits were procured from Yuanye Bio-Technology Co., Ltd. (Shanghai, China). The chemicals and reagents were procured from the National Pharmaceutical Group (Beijing, China).

### 2.2 Amylose content

Amylose content (AC) was determined using the colorimetric method described previously, with minor modifications (Zheng et al., 2024). Briefly, 0.1 g of rice powder was mixed with 1 mL of ethanol and 9 mL of 1 N NaOH solution, heated in a water bath at 90 °C for 20 min, and the volume adjusted to 100 mL with distilled water. Subsequently, 1 mL of 1 N acetic acid solution and 2 mL of 2% iodine solution were added to 5 mL of the above solution, and the mixture made up to 100 mL with distilled water. Finally, the mixture was incubated at 30 °C for 20 min, and then the absorbance was measured at 620 nm ultraviolet wavelength. The proportion of amylose is calculated by dividing amylose content by total starch content. The total starch content was determined by classical amylase enzymatic hydrolysis.

### 2.3 Expected glycemic index

The eGI of rice samples was determined according to the method described by Kaur et al. (2016), with minor modifications. Briefly, 0.5 g of rice flour was mixed with distilled water, gelatinized in a boiling water bath (99 °C) for 20 min, and then incubated at 37 °C with shaking at 170 rpm. A three-stage in vitro digestion was then performed by sequentially adding simulated digestive fluids under the same temperature and shaking speed: 10 mL simulated oral fluid for 15 min, 10 mL simulated gastric fluid for 1 h, and 10 mL simulated intestinal fluid for an additional 1 h.

During digestion, 0.5 mL digesta was collected at 0, 15, 45, 75, 105, 135, and 180 min, mixed with 2 mL ethanol, and centrifuged at 10000 ×*g* for 10 min. The glucose concentration in the supernatant was determined using the glucose oxidase-peroxidase method. The area under the curve (AUC) was calculated based on a glucose standard curve. The starch hydrolysis index (HI) and eGI were calculated from the AUC of glucose using Eqs. (1), (2) (Kunyanee et al., 2022).

(1) $$\mathrm{HI}\;\left(\%\right)=\frac{{\mathrm{AUC}}_{\text{sample}}}{{\mathrm{AUC}}_{\text{white bread}}}\times 100$$

(2) eGI=39.71+0.549HI

### 2.4 Gelatinization properties

The gelatinization properties of the rice samples were determined using a Rapid Viscosity Analyzer (RVA) (Li et al., 2016). In summary, 3.0 g of the rice sample was added to 25 mL of distilled water. The starch suspension was maintained at 50 °C for 1 min and subsequently heated to 95 °C at a rate of 12 °C/min. The reaction temperature was maintained at 95 °C for 2.5 min, after which the starch suspension was cooled to 50 °C at a rate of 12 °C/min. The suspension was left to stand at 50 °C for 2 min. Gelatinization parameters were recorded during the gelatinization process. The parameters of interest included gelatinization temperature, final viscosity, breakdown, and retrogradation.

### 2.5 Gel consistency

The gel consistency (GC) of the rice samples was determined according to the procedure described by Jiang et al. (2024), with minor modifications. Rice powder (100 mg) was placed in a glass tube, followed by the addition of 0.2 mL of 0.025% thymol blue solution. After thorough dispersion, 2 mL of 0.2 mol/L NaOH solution was added to the tube. The glass tube was heated in boiling water for 8 min and then transferred to ice water for cooling for 20 min. After cooling, the glass tube was placed horizontally in an incubator at 23 ± 2 °C and allowed to stand for 1 h. Finally, the length of the rice gel was measured.

### 2.6 Rice taste analysis

The taste value of cooked rice samples was determined by a rice taste analyzer (STA1B, Satake Corporation, Japan) (Zhu et al., 2022). Based on visible and near-infrared spectrophotometry, the instrument acquires reflection and transmission spectra of cooked rice samples and converts them into quantitative indices associated with rice appearance, texture and flavor via built-in algorithms. Polished rice (30.0 g) was weighed and distilled water was added at a ratio of 1:1.2. After soaking for 30 min, the mixture was steamed for 30 min, followed by stewing for 10 min, and then cooled to room temperature. 8 g of cooled rice was placed in a stainless-steel ring of the rice taste analyzer and pressed into a rice cake. The rice cake was then transferred to the measurement cell of the analyzer to determine the appearance score (AS), balance degree (BD), overall texture (OT), and taste value (TV) of the cooked rice. Subsequently, the rice cake was moved to the texture measurement cell to measure the hardness, elasticity, and viscosity of the cooked rice.

### 2.7 Sensory evaluation

Sensory evaluation of rice samples was conducted in accordance with the Chinese National Standard GB/T 15682–2008 (Fan et al., 2024). The sensory panel consisted of 10 graduate students and 5 teachers from the Department of Food Processing and Safety, Southwest Forestry University. They mainly evaluated the samples via tactile, visual, olfactory and gustatory perceptions. Prior to formal trials, all panelists received standardized special training for sensory evaluation to familiarize themselves with rice sensory indices, scoring criteria, sensory discrimination protocols and experimental precautions. Meanwhile, panelists were required to avoid spicy, pungent and strongly flavored foods, alcohol and tobacco within 24 h before each test to maintain normal sensory sensitivity. All sensory assessments were conducted in individual dedicated sensory booth rooms with uniformly controlled environmental conditions: ambient temperature of 22–25 °C, relative humidity of 50%–60%, and standard neutral balanced lighting.

After the rice samples were cooked, they were immediately dispensed into uniform, sterile, odor-free and clean dedicated experimental containers. The interval between the completion of cooking and sensory evaluation was controlled at 10 min, and the tasting temperature of samples was maintained at 55 ± 2 °C. The portion of cooked rice sample for each single assessment was 15 g. All cooked rice samples were randomly coded, and the evaluation sequence of samples for each panelist was randomly assigned. After finishing the evaluation of a single sample, panelists rinsed their mouths with pure water and rested for 30 s to 1 min to restore taste and olfactory perception before assessing the next sample.

Quantitative descriptive sensory analysis (QDA) was adopted for rice sensory evaluation in this study, and a 100-point quantitative scoring scale was established based on nine subdivided sensory attributes. The evaluation covered four attributes, namely rice appearance (color, gloss, and rice grain integrity), texture (cold rice texture, softness, flexibility, and adhesion), odor, and taste. The score weight of each indicator and detailed scoring criteria are shown in Table S2. Each panelist performed three randomized replicate evaluations for every sample. The score of each panelist for one sample was calculated as the average of the three replicates, while the final score of each sample represented the mean value from the 15 panelists. During statistical analysis, sensory panelists were treated as a random effect to eliminate systematic errors arising from individual differences in sensory discrimination habits.

### 2.8 Odor analysis by ultra-fast gas-phase electronic nose

The odor components of rice samples were analyzed using an Heracles NEO ultra-fast gas chromatography electronic nose (Alpha M.O.S., Toulouse, France) (Dai et al., 2024). This electronic nose is equipped with an automatic sampling device, ultra-fast gas chromatography (UF-GC), two flame ionization detectors (FID), and two chromatographic columns with different polarities (MXT-5 and MXT-1701). The Alphasoft data processing system combined with the exclusive AroChemBase flavor compound database supports signal acquisition of volatile components in samples and qualitative identification of corresponding compounds.

The acquisition time of the electronic nose for determining volatile components in rice was optimized based on a previous study (Li et al., 2023). The simplified procedure was as follows: First, 2.0 g of cooked rice sample was placed in a 10 mL headspace vial and equilibrated at 60 °C for 20 min. Subsequently, the headspace gas was extracted using a 2.5 mL gastight syringe (Hamilton, USA) and rapidly injected into the electronic nose system. The carrier gas flow rate was set at 150 mL/min, with a total acquisition time of 190 s and an acquisition interval of 0.5 s. All samples were analyzed in triplicate at different time points. o-Dichlorobenzene (0.094 μg/mL) was used as the internal standard. The relative content of each volatile compound in samples was calculated based on the ratio of the chromatographic peak area of each volatile component to that of the internal standard for relative quantification.

A mixture of n-alkanes ranging from C6 to C16 was used to calibrate the Kovats retention indices (RI) of the two chromatographic columns. The retention time of each chromatographic peak of the samples was converted into the corresponding retention index, which was then matched against the standard retention indices and mass spectrum information in the AroChemBase database. The relative odor activity value (ROAV) of odor compounds is calculated by Eq. (3). The random forest feature importance method was used to evaluate the importance of different odor compounds. Metabolites with mean decrease accuracy (MDA) values >1 were regarded as markers contributing significantly to sample classification (Liu et al., 2026).

(3) $${\mathrm{ROAV}}_{i}=100\times \frac{C_{i}}{C_{\max}}\times \frac{T_{\max}}{T_{i}}$$

where C is the relative concentration, T is the odor threshold, I is the target compound, and max is the compound with the largest odor activity value.

### 2.9 Untargeted metabolomics analysis

#### 2.9.1 Sample pretreatment

Metabolites extracted from rice grain samples were detected and analyzed following the reported ultra-high performance liquid chromatography-tandem mass spectrometry (UPLC-MS/MS) method (Zhu et al., 2022). Briefly, 100 mg of rice sample was accurately weighed and extracted with 1 mL methanol-water solution (volume ratio 4:1). The mixture was subjected to ultrasonic treatment for 20 min and then stood for 1 h. Afterwards, centrifugation was carried out at 4 °C and 16,000 g for 20 min, and the supernatant was concentrated to dryness by rotary evaporation. Prior to mass spectrometry analysis, the dried residue was reconstituted with 200 μL methanol-water solution (volume ratio 1:1), vortexed thoroughly, and centrifuged at 4 °C and 20,000 g for 15 min. The resulting supernatant was collected for subsequent UPLC injection analysis.

#### 2.9.2 Chromatography-mass spectrometry conditions

Chromatographic separation was performed on a Shimadzu LC-30 ultra-high performance liquid chromatography (UHPLC) system equipped with a Waters ACQUITY UPLC® HSS T3 column (2.1 mm × 100 mm, particle size 1.8 μm; Waters Corporation, Milford, MA, USA). The injection volume was 4 μL, the column temperature was maintained at 40 °C, and the flow rate was 0.3 mL/min. Mobile phase A consisted of aqueous solution containing 0.1% (*v*/v) formic acid, and mobile phase B was acetonitrile with 0.1% (*v*/v) formic acid. The gradient elution program was set as follows: 0–2 min, 0% B; 2–6 min, 48% B; 6–12 min, 100% B; 12–15 min, 0%. The flow rate was 0.3 mL/min, the column temperature was maintained at 40 °C, and the injection volume was 4 μL.

Mass spectrometric detection was carried out on a Thermo Fisher QE Plus Orbitrap mass spectrometer coupled with a heated electrospray ionization (HESI) source. Data acquisition was conducted under both positive (+) and negative (−) ionization modes. The spray voltage was set to 3.8 kV for positive mode and 3.2 kV for negative mode. Other universal parameters were configured as follows: capillary temperature, 320 °C; sheath gas flow rate, 30 arb; auxiliary gas flow rate, 5 arb; ion source heater temperature, 350 °C. The full scan resolution was 70,000 at *m*/*z* 200, and the resolution for data-dependent MS/MS (DDA-MS/MS) was 17,500. Stepped normalized collision energies of 20, 40 and 60 eV were applied for fragmentation.

#### 2.9.3 Data preprocessing and quality control

Raw mass spectrometry data were processed using MS-DIAL software (Version 4.9) to complete peak detection, peak alignment, retention time correction and peak area extraction. Pooled QC samples were prepared by mixing equal volumes of all rice test samples. One QC injection was inserted after every six analytical samples to monitor instrument stability. Metabolite features with relative standard deviation (RSD) < 30% across QC samples were retained, while features with RSD ≥ 30% were discarded as unstable signals to guarantee analytical reproducibility. One extraction blank (mobile phase only) was injected at the start and end of each batch run for background noise subtraction. Any feature whose signal intensity in blank samples exceeded three times the average intensity in actual rice samples was removed to eliminate interference from solvent and system contamination. Features with missing ion peaks accounting for more than 50% within any group (i.e., effective detection rate < 50%) were excluded from subsequent statistical analysis. Data from positive and negative ionization modes were separately normalized against the total peak area of each sample to eliminate variations in injection volume. All normalized datasets were preprocessed via Unit Variance Scaling to assign equal weight to all variables in subsequent pattern recognition analysis.

#### 2.9.4 Metabolite annotation and confidence level classification

Metabolite annotation in this study was accomplished by combining accurate mass matching with MS/MS spectral matching, and confidence levels were classified in accordance with the Metabolomics Standards Initiative (MSI) framework. No MSI Level 1 identifications were obtained owing to the absence of validation with authentic reference standards; only MSI Level 2 and Level 3 annotations were generated. MSI Level 2 (putatively annotated compounds) represented the highest annotation confidence tier in this work. Metabolites at this level required high matching consistency between precursor accurate masses, MS/MS fragmentation patterns and reference records from public databases (HMDB v5.0, MassBank v2023.11) as well as an in-house local database. The filtering thresholds were set as follows: precursor mass deviation ≤5 ppm, fragment ion mass tolerance ≤0.02 Da, and MS/MS dot product similarity score ≥ 70%. All auto-matched hits were manually curated to remove false positives. MSI Level 3 (tentative candidate compounds) referred to features merely preliminarily annotated via accurate mass matching, or those failing to meet the MS/MS matching thresholds without credible fragmental structural evidence, which were only retained as candidate substances for reference. To reduce false positive risks induced by multiple testing, the Benjamini–Hochberg method was applied for false discovery rate (FDR) correction, and metabolites with q-value <0.05 were defined as differential metabolites.

#### 2.9.5 Multivariate statistical analysis and screening of differential metabolites

The preprocessed normalized data matrix was imported into R software (Version 4.3.1). Principal component analysis (PCA) was performed to preliminarily visualize group separation trends and detect outlier samples. Supervised models were constructed using orthogonal partial least squares discriminant analysis (OPLS-DA). Differential metabolites were screened based on the combined criteria of variable importance in projection (VIP) > 1.0 and FDR-adjusted q-value <0.05 as described above.

### 2.10 Statistical analysis

Microsoft Excel, IBM SPSS Statistics V.25.0 and Origin 2018 were used for basic data sorting and plotting. Three biological replicates were set for each rice sample, and the results were expressed as mean ± SD. Multivariate pattern recognition analyses were implemented in R software (Version 4.3.1). Prior to univariate intergroup comparisons, normality and homogeneity of variance tests were performed on the data. Duncan's multiple range test was subsequently adopted, and differences with *P* < 0.05 were considered statistically significant. Pearson correlation analysis was performed using IBM SPSS Statistics V.25.0. The Pearson correlation coefficient was adopted to quantify the degree of linear correlation between different indicators.

## 3 Results

### 3.1 Amylose content and eGI value

Amylose content and its proportion are one of the key factors that determine the cooking quality and digestive characteristics of rice (Wang et al., 2023). As shown in Figs. 1A-B, YNUR exhibited relatively high levels of amylose content (11.78–14.11%) and proportion (15.19–18.45%). Among them, LC had the highest amylose content and the largest proportion of total starch. Jiangxi paddy rice (JXPR) exhibited the lowest amylose content and proportion, whereas Heilongjiang paddy rice (HLJPR) showed intermediate levels. Wang et al. (2023) also found that the amylose content of upland rice was higher than that of paddy rice. The eGI value is used to evaluate the ease of starch hydrolysis in foods, which are not equivalent to actual in vivo glycemic responses (Kaur et al., 2016). As illustrated in Fig. 1C, YNUR exhibited relatively low eGI values (60.55–69.13), with KY having the lowest eGI value. HLJPR showed the highest eGI values, with DHX reaching approximately 79.55, while JXPR fell in between (70.11–74.96). Overall, the eGI values of YNUR were significantly lower than those of JXPR and HLJPR (*P* < 0.05).

**Fig. 1:**
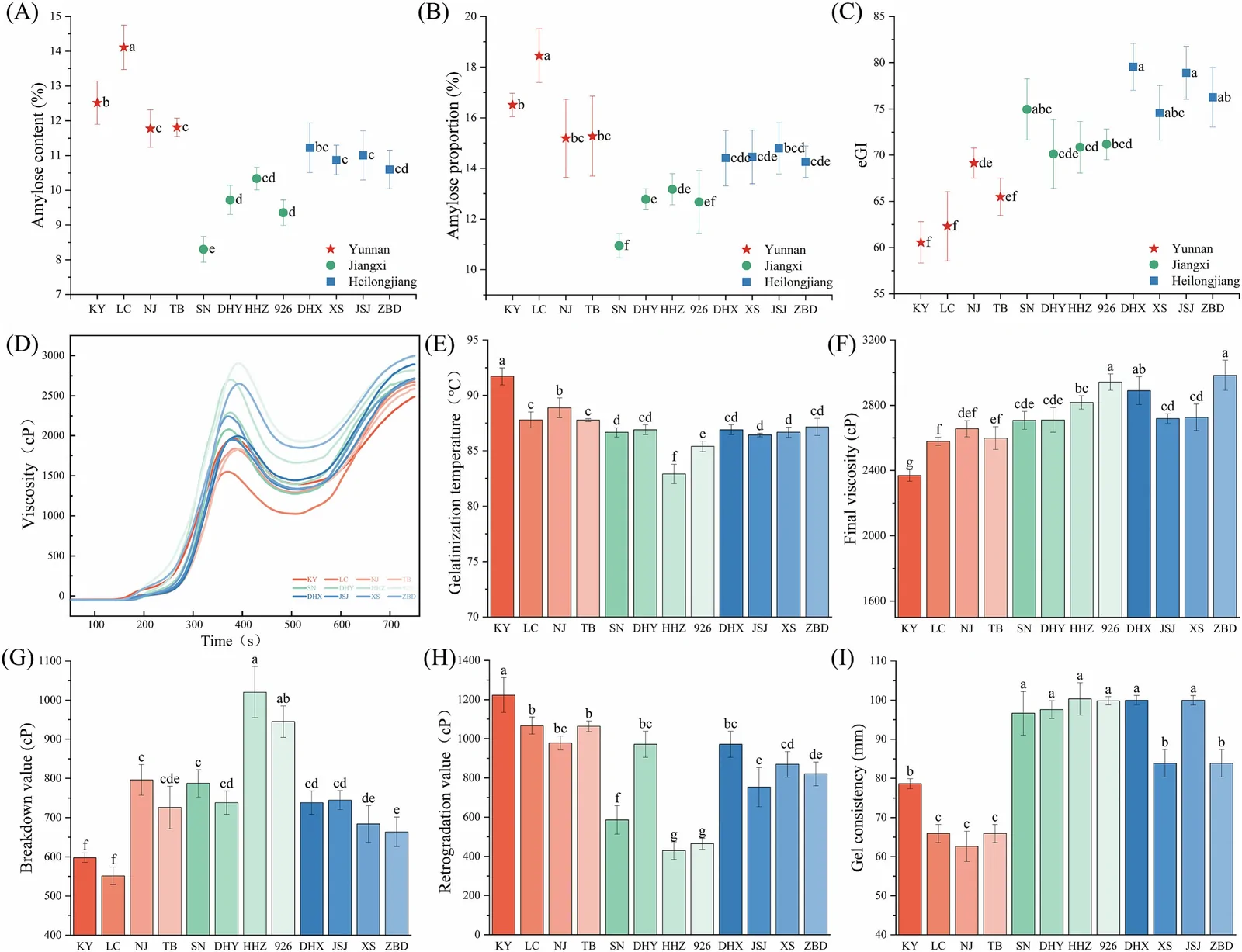
Amylose content (A), amylose proportion (B) and eGI value (C) of different rice samples. Gelatinization curves of different rice samples (D). E-I represent gelatinization temperature, final viscosity, breakdown value, retrogradation value, and gel consistency, respectively. The results were expressed as mean ± SD.

### 3.2 Gelatinization properties

The gelatinization curves of different rice samples were obtained using RVA, as shown in Fig. 1D. The viscosity changes presented in the gelatinization curves allowed for the calculation of various gelatinization parameters (Feng et al., 2025). As shown in Fig. 1E, the gelatinization temperature of YNUR was significantly higher than that of JXPR and HLJPR (*P* < 0.05), with KY exhibiting the highest gelatinization temperature (nearly 92.00 °C). This indicates that starch in YNUR had stronger thermal stability. Final viscosity, which reflects the viscosity change of starch during the cooling phase after gelatinization (Jannasch & Wang, 2025). Fig. 1F shows that the four paddy rice from Heilongjiang had the highest final viscosity (2719.33–2984.33 cP), followed by the paddy rice from Jiangxi (2707.67–2942.33 cP). In contrast, YNUR generally exhibited lower final viscosity, with KY at 2369.00 cP.

Breakdown value indicates the viscosity stability of gelatinized starch under high-temperature shear, retrogradation value and gel consistency collectively reflect the hardness and rigidity of cooked rice after cooling (Feng et al., 2025; Jannasch & Wang, 2025). As shown in Fig. 1G, the breakdown value of YNUR was significantly lower than that of the other two paddy rice groups (*P* < 0.05). The breakdown values of KY and LC were both below 600.00 cP. YNUR showed relatively high retrogradation value and low gel consistency, among which KY had the highest retrogradation value (1222.67 cP) and the lowest gel consistency (100.33 mm) (Fig. 1H). This indicated that YNUR became harder than lowland rice after cooling. Wang et al. (2025) reported that compared with paddy rice, the upland rice Han 9710 had a higher gelatinization temperature and lower final viscosity, which is consistent with the results of this study. However, unlike our findings, upland rice Han 9710 had a higher breakdown value than paddy rice.

### 3.3 Taste characteristics

A rice taste analyzer was used to measure the taste parameters of different rice samples (Table 1). Appearance score (AS) quantified the visual quality of cooked rice in terms of grain integrity, color, glossiness, and fluffiness (Feng et al., 2025). YNUR exhibited intermediate appearance scores, with TB and LC scoring 8.53 and 8.00, respectively, which were comparable to or even better than some paddy rice varieties, such as JXPR 926 (6.70), DHY (7.40), SN (8.13), and HLJPR ZBD (8.00). This indicated that YNUR outperformed certain paddy rice varieties in terms of grain integrity and color. Taste value (TV) comprehensively evaluated the flavor quality of cooked rice based on umami, sweetness, and kokumi (Wang et al., 2023). YNUR (8.75–9.01) scored significantly higher than JXPR (6.90–7.88) and HLJPR (7.93–8.20) (*P* < 0.05), with NJ achieving the highest taste value (9.01). This suggested that YNUR exhibited more intense umami and sweetness.

**Table 1:** Taste characteristics of different rice varieties.

Rice type	Appearance	Hardness	Elasticity	Viscosity	Overall Texture	Taste Value	Balance degree
KY	7.33 ± 0.12^fg^	4.84 ± 2.26^ab^	0.79 ± 0.04^a^	0.32 ± 0.05^b^	7.10 ± 0.20^f^	8.75 ± 0.44^a^	0.09 ± 0.00^bcd^
LC	8.00 ± 0.10^def^	4.40 ± 0.73^ab^	0.77 ± 0.03^ab^	0.34 ± 0.11^ab^	7.47 ± 0.35^def^	8.80 ± 0.26^a^	0.08 ± 0.01^cd^
NJ	7.87 ± 0.06^def^	5.50 ± 1.06^a^	0.79 ± 0.01^a^	0.35 ± 0.09^ab^	7.87 ± 0.23^cde^	9.01 ± 0.31^a^	0.11 ± 0.01^bcd^
TB	8.53 ± 1.01^bcd^	4.95 ± 0.38^ab^	0.76 ± 0.03^ab^	0.42 ± 0.07^ab^	7.30 ± 0.10^ef^	8.82 ± 0.12^a^	0.10 ± 0.04^bcd^
SN	8.13 ± 0.25^cde^	3.58 ± 0.36^b^	0.71 ± 0.03^c^	0.55 ± 0.05^a^	8.70 ± 0.26^b^	7.43 ± 0.38^cd^	0.09 ± 0.03^bcd^
DHY	7.40 ± 0.40^efg^	4.24 ± 0.50^ab^	0.74 ± 0.01^abc^	0.42 ± 0.16^ab^	8.93 ± 0.76^ab^	7.32 ± 0.10^de^	0.12 ± 0.02^abc^
HHZ	8.90 ± 0.70^b^	4.58 ± 0.53^ab^	0.74 ± 0.02^abc^	0.47 ± 0.01^ab^	8.20 ± 0.10^bcd^	6.90 ± 0.18^e^	0.08 ± 0.02^cd^
926	6.70 ± 0.17^g^	4.20 ± 0.17^ab^	0.76 ± 0.02^ab^	0.55 ± 0.29^a^	7.90 ± 0.10^cde^	7.88 ± 0.08^bc^	0.08 ± 0.03^cd^
DHX	8.80 ± 0.17^bc^	3.89 ± 0.27^ab^	0.73 ± 0.06^bc^	0.56 ± 0.03^a^	8.27 ± 0.25^bc^	8.20 ± 0.25^b^	0.14 ± 0.01^a^
JSJ	8.90 ± 0.26^b^	4.21 ± 0.25^ab^	0.76 ± 0.01^ab^	0.50 ± 0.06^ab^	8.43 ± 1.01^bc^	8.15 ± 0.44^b^	0.12 ± 0.02^abc^
XS	9.73 ± 0.06^a^	4.57 ± 0.31^ab^	0.78 ± 0.01^ab^	0.44 ± 0.05^ab^	8.83 ± 0.06^ab^	8.10 ± 0.10^b^	0.13 ± 0.02^ab^
ZBD	8.00 ± 0.26^def^	4.06 ± 0.34^ab^	0.77 ± 0.02^ab^	0.42 ± 0.13^ab^	9.50 ± 0.00^a^	7.93 ± 0.25^b^	0.12 ± 0.01^ab^

Notes: Different lowercase letters within the same column indicate significant differences among different samples (*P* < 0.05). The results were expressed as mean ± SD.

A rice taste analyzer evaluated the mastication and orosensory perception of cooked rice through texture parameters. The hardness of YNUR (4.40–5.50) was higher than that of JXPR (3.58–4.58) and HLJPR (3.89–4.57), indicating that YNUR had a firmer texture (Wang et al., 2025). In terms of elasticity, there was no significant difference between YNUR (0.76–0.79) and HLJPR (0.73–0.78), but both were slightly higher than JXPR (0.71–0.76), suggesting that YNUR exhibited better recovery during chewing. Notably, the viscosity of YNUR (0.32–0.42) was generally lower than that of JXPR (0.42–0.55) and HLJPR (0.42–0.56), indicating that the viscosity between YNUR grains was relatively low and they were not prone to caking. Balance degree served as a comprehensive indicator for assessing the harmony between flavor and texture, reflecting the palatability of cooked rice (Feng et al., 2025). HLJPR exhibited relatively high balance degrees (0.12–0.14), while no significant differences were observed among YNUR (0.08–0.11) and JXPR (0.08–0.12) (*P* > 0.05), indicating that the palatability of YNUR was comparable to that of JXPR. In summary, YNUR exhibited drawbacks including excessive hardness and moderate appearance quality, while featuring low viscosity, high elasticity, and high flavor perception intensity.

### 3.4 Sensory evaluation

To further evaluate the sensory quality of different rice samples, this study conducted sensory evaluations of all cooked rice from three aspects (Zhou et al., 2023). Fig. S1 presented the sensory evaluation results of the 12 rice samples. In terms of appearance quality, the grain integrity scores of YNUR ranged from 3.57 to 4.07, which were significantly higher than those of HLJPR (3.29–3.93) and JXPR (3.14–3.71). No significant differences were observed in gloss among the three rice groups (*P* > 0.05), but the color scores of YNUR (4.43–5.34) were generally lower than those of JXPR (5.14–6.16) and HLJPR (4.86–6.00). Regarding texture quality, the softness scores of YNUR ranged from 6.57 to 6.64, slightly lower than those of the other two rice groups, although the differences were not significant (*P* > 0.05). YNUR exhibited excellent flexibility scores (5.79–7.43), with LC achieving a flexibility score of 7.43, comparable to that of Heilongjiang paddy rice. The adhesion scores of YNUR ranged from 5.93 to 6.64, which were significantly lower than those of JXPR (6.07–7.29) (*P* < 0.05), indicating that YNUR grains were distinct and less prone to sticking. In terms of cooled rice texture, YNUR scored relatively low (3.14–3.79), suggesting a slight firming of texture upon cooling. In terms of flavor quality, the odor scores of YNUR ranged from 16.07 to 17.07, significantly higher than those of JXPR (14.00–15.14) and comparable to those of HLJPR (14.21–17.64). YNUR demonstrated outstanding taste scores, ranging from 17.50 to 21.86, with LC achieving a taste score of 21.86, significantly higher than that of most rice varieties. This indicated that YNUR had distinct advantages in terms of strong flavor, aroma persistence, and taste complexity.

### 3.5 Odor characteristics

#### 3.5.1 Odor fingerprint

As shown in Fig. 2A-B, the results obtained using the MXT-5 and MXT-1701 columns exhibited similar trends; however, their separation capabilities for different volatile components varied owing to differences in polarity. In the study by Ai et al. (2025), the separation performance of the two chromatographic columns was similar to that obtained in this study. A total of 160 odor-active compounds were tentatively identified in 12 rice samples by matching mass-spectrometric information with standard retention indices from databases. More volatile compounds were detected in YNUR than in paddy rice, and the NJ sample had the highest number, with 90 identified compounds. For instance, aldehydes such as phenylacetaldehyde and hexanal, as well as esters including ethyl acetate and methyl butyrate, were exclusively detected in YNUR. In addition, the relative abundance of hexanal in YNUR was 1.2–1.5 times that in paddy rice. These compounds may be related to pleasant aromas such as fresh and fruity notes, and are key contributors to the intense aroma of cooked YNUR (Wongsa et al., 2025).

**Fig. 2:**
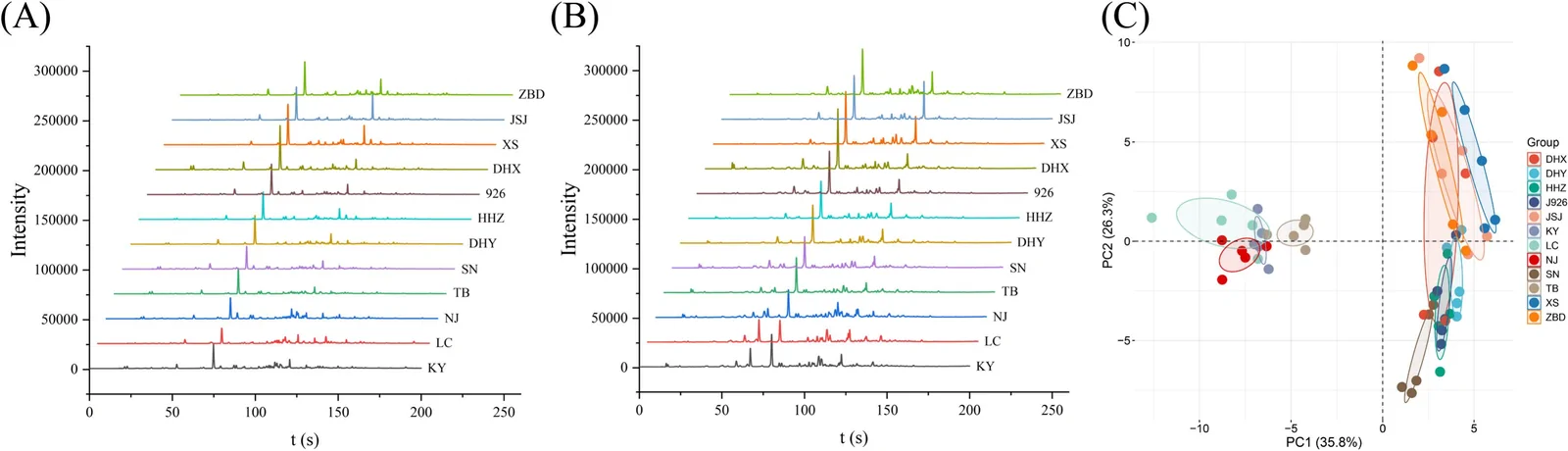
Gas chromatogram overlay diagrams obtained by MXT-5 (A) and MXT-1701 (B) columns. PCA cluster analysis based on the relative content of odor compounds in rice samples (C).

Principal component analysis (PCA) was performed based on the relative content of all odor compounds (Fig. 2C). YNUR samples were primarily concentrated in the left region and were clearly separated from JXPR and HLJPR. JXPR samples were mainly distributed in the upper-right region, and HLJPR samples were predominantly clustered in the lower-right region, suggesting distinct geographical origin- and variety-specific volatile profiles for the three rice types. The YNUR samples exhibited a clear clustering trend in the plot, indicating a high consistency in their volatile composition and significant differences from the two types of paddy rice.

#### 3.5.2 Differential odor compounds

Based on the detection results of the Heracles NEO ultra-fast gas chromatography electronic nose, odor compounds were screened using the discrimination power function embedded in the software. Chromatographic peaks with a discrimination power > 0.800, peak area > 100, and satisfactory separation were defined as differential odor compounds (Zhang et al., 2022). A total of 15 differential compounds were screened from all samples, and their putative odor descriptions and odor thresholds are shown in Table S3 and Fig. 3A. These differential compounds were classified into six categories: aldehydes, ketones, terpenes, aromatic hydrocarbons, fatty hydrocarbons, and heterocycles. As shown in Fig. 3B, YNUR exhibited high levels of aldehydes, ketones, aromatic hydrocarbons, and fatty hydroarbons, which provided an important material basis for its intense aroma characteristics. Notably, toluene, naphthalene, pyridine and styrene were detected in partial samples. These compounds may originate from environmental background, packaging materials or instrumental contamination, and were therefore excluded from subsequent differential analysis.

**Fig. 3:**
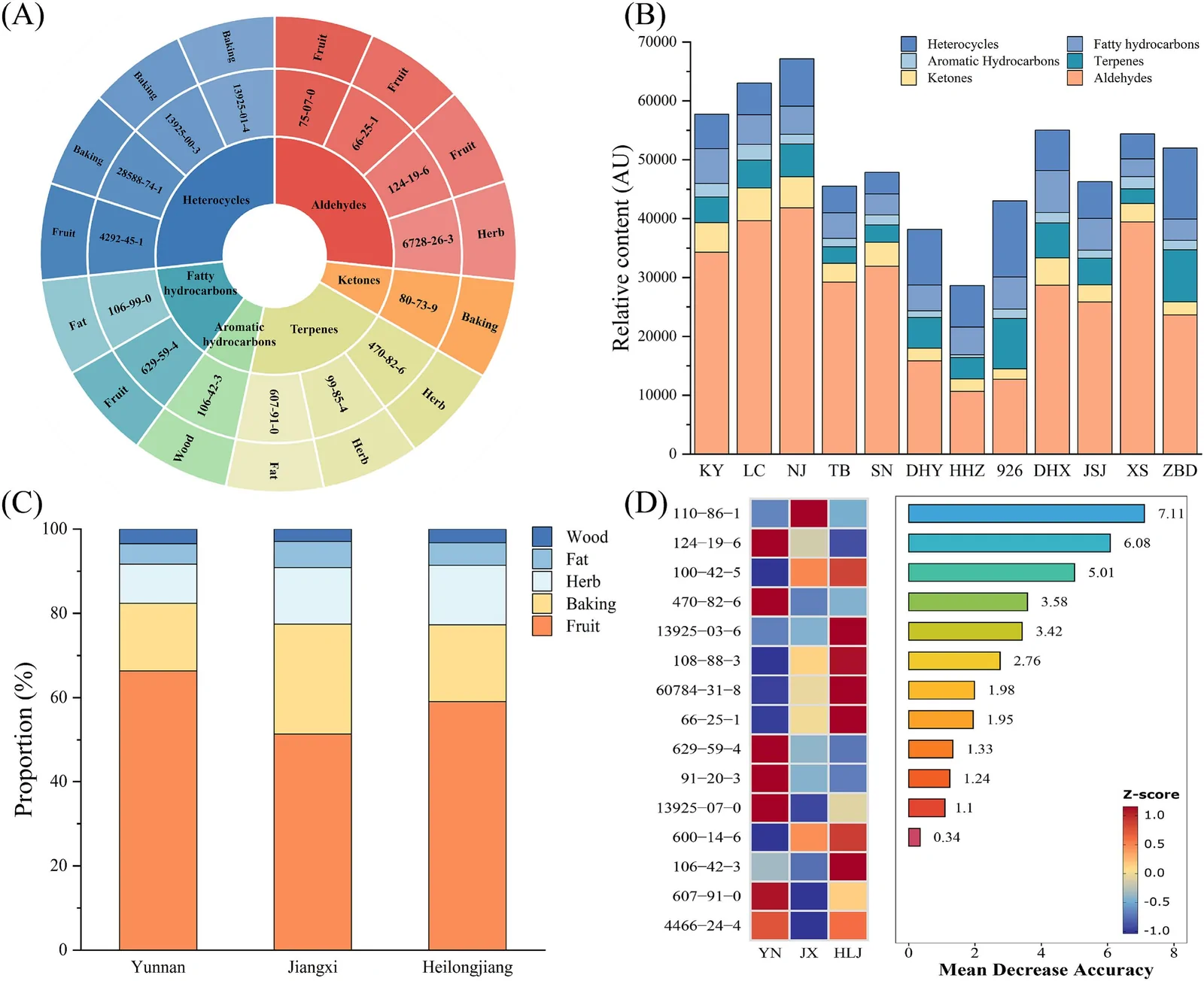
Information wheel diagram of different odor compounds (A), relative content distribution diagram of different kinds of different compounds (B), proportion of compounds with different odor descriptions in rice samples from different regions (C), and random forest analysis result diagram based on mean decrease accuracy (MDA) (Only 12 compounds obtained MDA values) (D).

To further compare the differences in odor profiles among different rice varieties, 15 distinct odor compounds were preliminarily classified into five categories based on sensory descriptions reported in the literature: fruity, roast, herb-like, fatty, and woody (Wongsa et al., 2025; Qi, Ren, Su, et al., 2026). The relative proportions of each odor category in YNUR, JXPR, and HLJPR were analyzed (Fig. 3C). YNUR exhibited the highest proportion of fruity odor (66.35%), which was significantly higher than that of JXPR (51.33%) and HLJPR (59.05%) (*P* < 0.05). The fruity odor was mainly contributed by acetaldehyde, hexanal, nonanal, (Z)-2-nonenal, and tetradecane, which may impart a fresh and sweet fruity characteristic to cooked rice (Wang et al., 2026). The proportion of herb-like in YNUR was 9.29%, respectively, which was lower than that in JXPR (13.40%) and HLJPR (14.13%). Herb-like odors are primarily derived from aldehydes, and terpenes, such as (Z)-2-nonenal, gamma-terpinene, and 1, 8-cineole (Ren et al., 2026). Compared with YNUR, the two paddy rice varieties exhibited significantly higher proportions of baking odor (*P* < 0.05), accounting for 26.11% and 18.23%, respectively. The roast odor compounds mainly consisted of 2,3-pentanedione, 2-methyl-3-furanthiol, 2-ethyl-6-methylpyrazine, and 2-ethyl-3,5-dimethylpyrazine (Qi, Ren, Yang, et al., 2026). Therefore, YNUR was rich in compounds defined as fruity, with relatively fewer Herb-like odor compounds.

#### 3.5.3 Key characteristic Odor compounds

The random forest feature importance method was used to evaluate the feature importance of the 15 differential odor compounds. The analysis results of the random forest feature importance method revealed that multiple compounds exhibited mean decrease accuracy (MDA) values >1, demonstrating their substantial contribution to sample classification (Liu et al., 2026). Only 12 odor compounds obtained MDA values, as shown in Fig. 4D. The results showed that 11 compounds had MDA values greater than 1, namely Pyridine, N-nonanal, Vinylbenzene, 1,8-Cineole, 2-Ethyl-6-methylpyrazine, Toluene, (Z)-2-nonenal, Hexanal, Tetradecane, Naphthalene, and 2-Ethyl-3,5-dimethylpyrazine, demonstrating their substantial contributions to sample classification. By combining the ROAV values and odor descriptions of the corresponding compounds listed in Table S2, the key compounds playing dominant roles in the flavor profiles were further identified. (Z)-2-nonenal and nonanal, which exhibit strong green and fruity aromas, showed significantly higher ROAV values in YNUR samples than in most paddy rice samples, along with high MDA values (Wang et al., 2026). Additionally, 2-ethyl-3,5-dimethylpyrazine generally had high ROAV values in YNUR, and its MDA was greater than 1, contributing to the roasted aroma of rice (Zhang et al., 2025). Based on the screening criteria requiring both MDA and ROAV values >1, (Z)-2-nonenal, nonanal, and 2-ethyl-3,5-dimethylpyrazine were identified as key characteristic odor compounds distinguishing YNUR from JXPR and HLJPR.

**Fig. 4:**
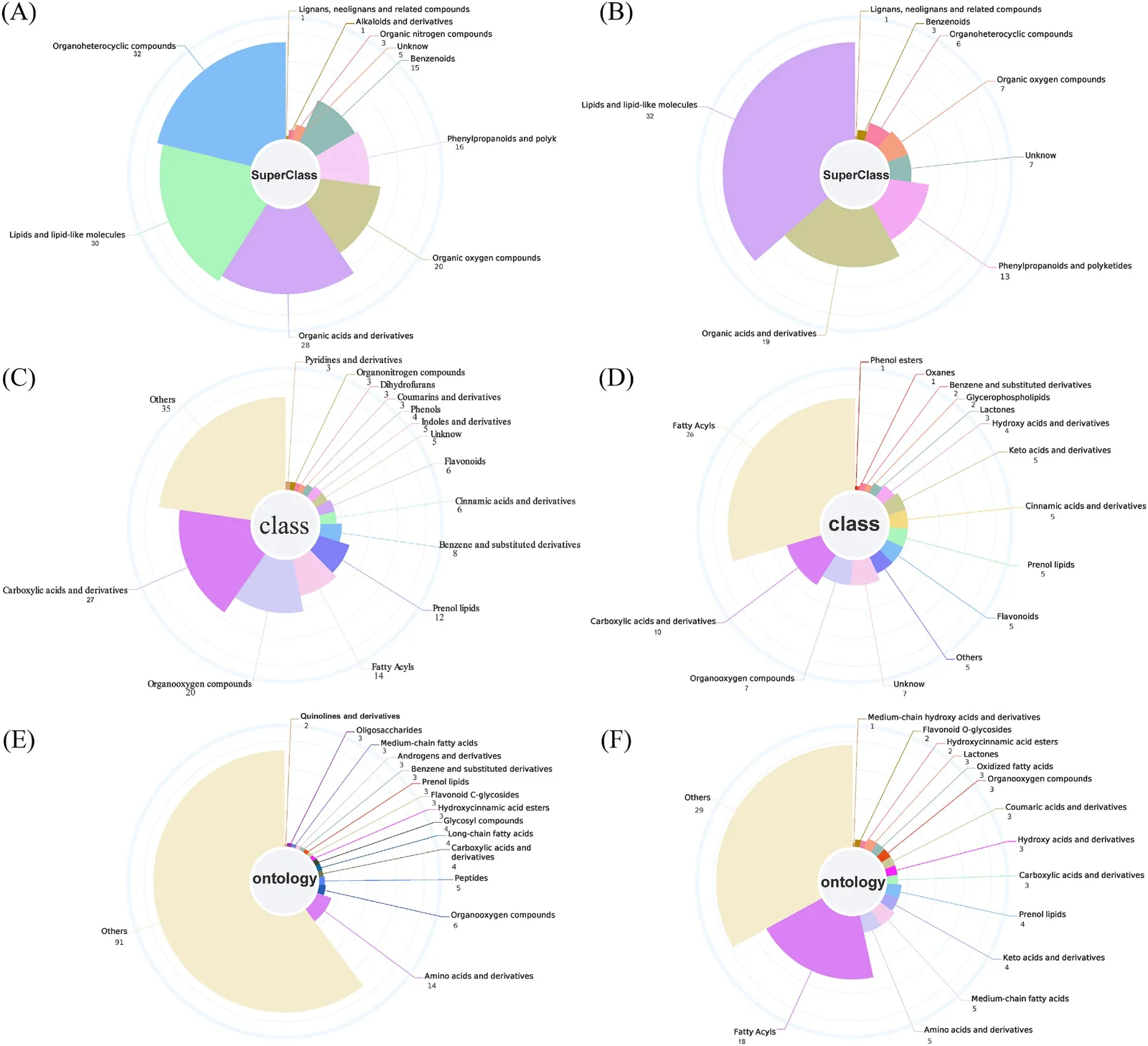
Schematic diagrams of metabolite classification in rice samples under positive ionization modes (A, C, and E) and negative ionization modes (B, D, and F). Different colors represent distinct categories and the sector area reflects the relative proportion of the corresponding metabolite category.

### 3.6 Untargeted metabolomics analysis

#### 3.6.1 Metabolic profile of rice samples

To reveal the differences in metabolite composition between upland and paddy rice samples, untargeted metabolomics analysis was performed on 12 rice samples (Cheng et al., 2023). A total of 1240 metabolites were detected, of which 717 were successfully matched in the positive ion mode and 523 in the negative ion mode. The composition and classification of metabolites are presented as pie charts. As shown in Figs. 4A-F, the primary metabolites of rice samples included organic heterocyclic compounds, lipids and lipid-like molecules, organic acids and derivatives, and organic oxygen compounds. At the class level, the metabolites principally include carboxylic acids and derivatives, organic oxygen compounds, fatty acyls, and prenol lipids.

#### 3.6.2 Multivariate statistical analysis

To analyze the metabolomic differences among various rice germplasms, unsupervised PCA was performed to reduce the dimensionality of the metabolomic data (Xiong et al., 2022). The results showed that PC1 (30.9%) and PC2 (23.9%) together explained 54.8% of the metabolomic variation (Fig. 5A), which was insufficient to discriminate differences among samples. Therefore, OPLS-DA was applied to further enhance the separation tendency of samples (Zhao, Xi, Xu, Jin, et al., 2022). On the t1(49.70%) and to1(32.60%) axes, YNUR formed a tight and independent cluster, which was obviously different from JXPR and HLJPR, indicating that its metabolic characteristics have high intra-group consistency and inter-group uniqueness (Fig. 5B). The permutation test was performed to verify the overfitting of the PLS-DA model. The intercepts of R^2^ and Q^2^ were 0.8900 and − 0.7888, respectively (Fig. 5C). The intercept of Q^2^ was less than 0, indicating that the PLS-DA model had no overfitting and exhibited good predictive ability.

**Fig. 5:**
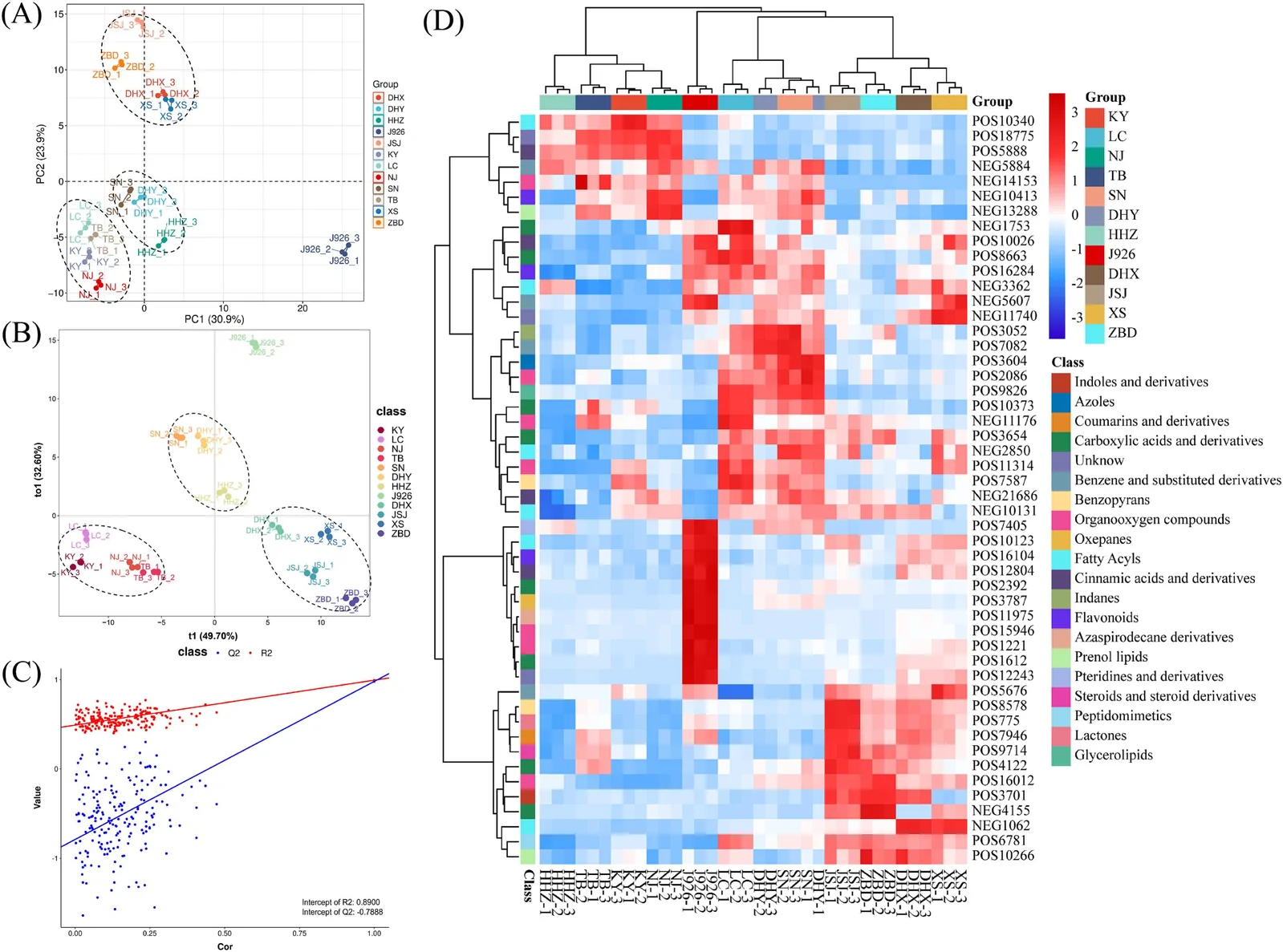
Multivariate statistical score of different rice samples. (A) PCA, (B) OPLS-DA, (C) OPLS-DA permutation testing, (D) Cluster heat map constructed by the top 50 different metabolites.

#### 3.6.3 Identification of differential metabolites

Based on the screening criteria of FDR-adjusted q-value <0.05 and OPLS-DA model (VIP > 1.0), 351 differential metabolites were identified in the positive ion mode and 222 in the negative ion mode (Cheng et al., 2023). The top 50 differential metabolites with the most significant abundance changes were selected to construct a clustering heatmap, thereby elucidating the metabolic differentiation characteristics among rice accessions from Yunnan, Jiangxi, and Heilongjiang. As shown in Fig. 5D, the samples in each group exhibited good intra-group repeatability, which verified the stability and reliability of the metabolomic differences between the groups (Zhang et al., 2023). The four YNUR varieties were tightly clustered into one branch, and the two branches formed by JXPR and HLJPR belonged to the same main branch, indicating that the metabolic characteristics of YNUR were significantly different from those of JXPR and HLJPR.

Based on the K-means clustering algorithm, all the differential metabolites were classified into six clusters, and Clusters 1 to 6 contained 162, 7, 90, 60, 72, and 113 metabolites, respectively (Fig. 6) (Yu et al., 2022). In terms of cluster size and functional enrichment, Cluster 1 and Cluster 6 were dominated by metabolites involved in basic metabolism (e.g., carbohydrates, amino acids), with relatively low expression differences among groups. Cluster 4 and Cluster 5 were enriched in numerous specific metabolites associated with environmental adaptability. Notably, metabolites in Cluster 4 exhibited high abundance in YNUR, while they were expressed at low levels in Jiangxi and HLJPR samples.

**Fig. 6:**
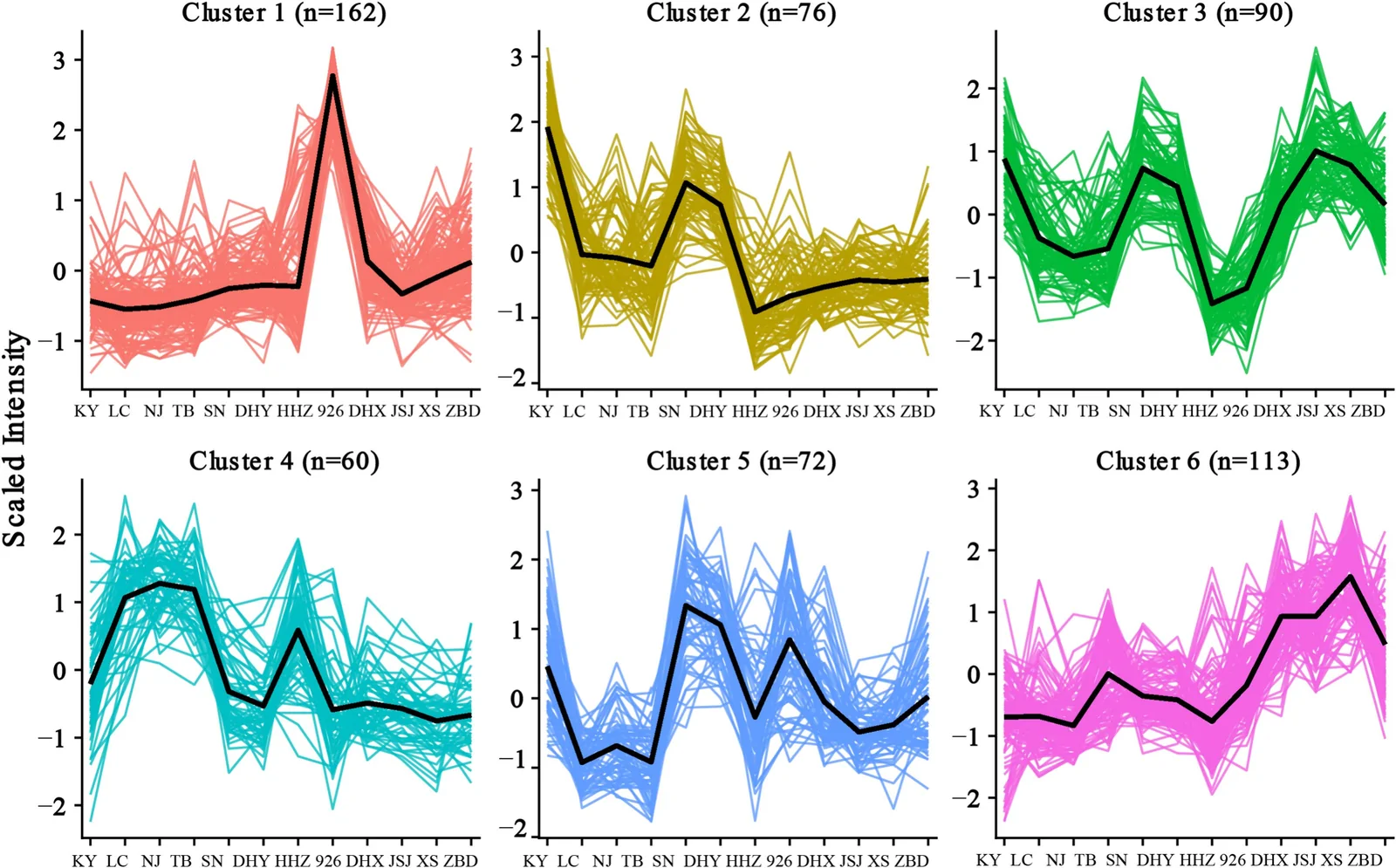
Six model expression profiles of rice samples based on K-means clustering calculation.

#### 3.6.4 Enrichment analysis of amino acid metabolic pathways

Amino acids are the core nutrients of rice and one of the key factors determining its protein nutritional value, flavor, and processing quality (Heo et al., 2026). To investigate the functional differentiation of amino acid metabolism among different rice varieties, KEGG pathway enrichment analysis was performed on 25 differential amino acids, and the results are shown in Fig. 7. The Sankey diagram (Fig. 7A) showed that these amino acids were mainly enriched in four metabolic modules: biosynthesis of amino acids, arginine and proline metabolism, cyanoamino acid metabolism, and D-amino acid metabolism (Luo et al., 2024). Among them, “biosynthesis of amino acids” was the central enriched pathway (orange module), covering 18 amino acids, indicating its core role in the regulation of amino acid metabolism in rice. The metabolic network correlation diagram further verified these results. As shown in Fig. 7B, the green nodes (core amino acids) displayed the densest connections with the “biosynthesis of amino acids” pathway, implying that this pathway acts as a key hub driving the diversity of amino acid metabolism in rice (Heo et al., 2026).

**Fig. 7:**
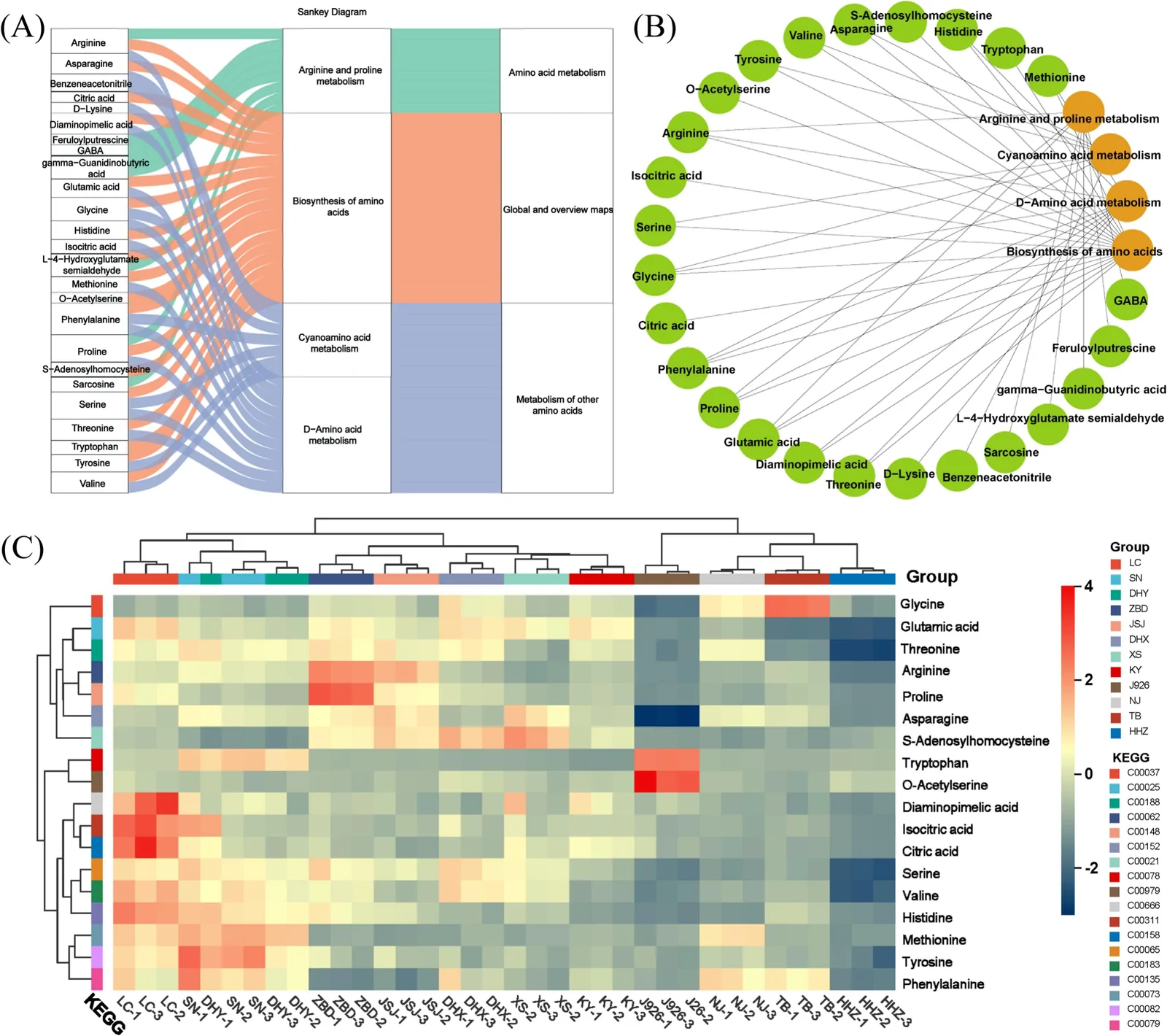
(A) Enrichment correlation analysis of KEGG pathways. (B) KEGG pathway network diagram, in which orange elliptical nodes represent pathways and green ones represent metabolites. (C) Heatmap showing the abundance distribution of 18 amino acids in different rice samples. (For interpretation of the references to color in this figure legend, the reader is referred to the web version of this article.)

The heatmap intuitively shows the abundance distribution patterns of the 18 amino acids in different rice varieties (Fig. 7C). Clustering analysis revealed significant differentiation in the amino acid profiles among the sample groups. YNUR exhibited an overall high-abundance amino acid profile, characterized by significantly up-regulated levels of glycine, glutamic acid, threonine, and arginine (concentrated red blocks). The amino acid profile of JXPR was relatively uniform, with most amino acids at the middle level, and only phenylalanine and tyrosine showed slight upregulation. HLJPR showed an overall low amino acid abundance, especially proline, asparagine, and S-adenosylhomocysteine were significantly down-regulated (concentrated blue blocks).

## 4 Discussion

### 4.1 Quality characteristics of YNUR

The results of this study indicate that the linear molecular structure of high-amylose starch (Figs. 1A-B) in YNUR facilitates the formation of dense and ordered spatial configuration of starch granules (Pérez-Santos et al., 2026), as reflected by the higher gelatinization temperature of YNUR (Fig. 1E), which renders the starch granules more resistant to structural disintegration during cooking (Theanjumpol et al., 2020). Additionally, this structural feature significantly hinders the thermal gelatinization and enzymatic hydrolysis of starch, thereby delaying its in vitro hydrolysis rate. This may be the key structural mechanism of YNUR's lower eGI (Fig. 1C) (Ngo et al., 2024).

The high retrogradation value reflected the tendency of YNUR starch to retrograde after cooling, which explained the higher hardness and lower gel consistency observed in cooked YNUR upon cooling (Fig. 1I) (Feng et al., 2025). Additionally, correlation analysis revealed that retrogradation value was positively correlated with hardness and negatively correlated with gel consistency (*P* < 0.05) (Fig. S2). Sensory evaluation and instrumental measurements demonstrated that YNUR had the disadvantage of excessive hardness leading to poor chewability, while its advantages included low adhesiveness, high elasticity and strong aromatic flavor (Table 1 and Fig. S1). Accordingly, YNUR may appeal to consumers who favor intense rice flavor, and is also suitable for processing into food with unique flavor.

Compared with paddy rice, YNUR had a richer variety of volatile compounds (Section 3.5.1), with higher contents of aldehydes, ketones, aromatics, and aliphatic hydrocarbons (Fig. 3B), especially aldehydes, such as nonanal, which contribute to fruity aromas (Table S3). Combined with the results of sensory evaluation (Fig. S1), the aroma profile of YNUR was identified to be dominated by fruit and baking aroma. Consistently, the key aroma compounds screened based on MDA values, namely hexanal, nonanal and 2-ethyl-3,5-dimethylpyrazine, were reported to have the aroma of fruit and baking (Wang et al., 2026; Zhang et al., 2025). Therefore, these compounds may serve as inter-group differential markers for YNUR grown under rainfed upland conditions, which can distinguish it from paddy rice cultivated under flooded conditions. Zhao, Xi, Xu, Yin, et al. (2022) also identified nonanal as a key odor-active compound for distinguishing rice varieties with different aroma types.

### 4.2 Metabolic characteristics of YNUR

In this study, the essential differences in metabolite composition between YNUR and JXPR or HLJPR were systematically revealed using non-targeted metabolomics combined with multivariate statistical analysis. For instance, the abundance of glycerophospholipids in YNUR was 1.8–2.1 times higher than that in paddy rice. K-means clustering divided the differential metabolites into six functional clusters, among which 60 metabolites in Cluster 4 showed a YNUR-preferred expression pattern, that is, they accumulated in high abundance in YNUR but were generally low expressed in rice. Functional annotation showed that metabolites in this cluster were highly enriched in three major stress-resistant functional modules: glycerophospholipid metabolites, stress signaling molecules, and osmoregulatory organic acids (Liu et al., 2025; Xiong et al., 2025). Glycerophospholipid metabolites have not previously been reported in upland rice research. Xiong et al. (2025) investigated the metabolome-based physiological response mechanism of *B. chinensis* under drought stress and proposed that stress-tolerant metabolites (such as glycerophospholipids) correlate with membrane lipid unsaturation and structural stability of lipid raft domains, which may influence cell membrane integrity and function under drought and dehydration.

Targeted analysis of amino acid metabolism pathways can reveal the accumulation and transformation of amino acids in different rice varieties during growth. KEGG enrichment analysis showed that 25 differential amino acids were mainly enriched in three pathways: “biosynthesis of amino acids”, “arginine and proline metabolism”, and “cyanoamino acid metabolism” (Figs. 7A-B). The amino acid biosynthesis pathway was at the core of the network (covering 18 amino acids), indicating that amino acid metabolism in YNUR was regulated by this pathway. Heatmap analysis showed that YNUR had a high-abundance amino acid profile, characterized by the significant upregulation of glycine, glutamic acid, threonine and arginine, while the overall amino acid abundance of HLJPR was low, and JXPR was at an intermediate level (Fig. 7C). These amino acids are reported for the first time to accumulate at high abundance in plateau upland rice. Rautela et al. (2026) proposed that osmoprotectants including proline, glycine betaine and sugars constitute key biochemical mechanisms underlying crop drought tolerance.

The correlation between metabolites and quality indicators of different rice varieties was established by correlation analysis. Glycerophospholipids significantly enriched in YNUR were positively correlated with amylose content (*P* < 0.05) (Fig. S2). Recent studies indicate that metabolic intermediates of glycerophospholipids are associated with the translation of starch-synthesizing enzymes, which may partly explain the higher amylose content in upland rice (Liu et al., 2025; Xiong et al., 2025). In terms of sensory quality, both the taste value and taste sensory scores of YNUR exhibited significant positive correlations with glycine and glutamic acid, and glycerophospholipids showed significant negative correlations with adhesiveness and breakdown value (*P* < 0.05) (Fig. S2). Glycine and glutamic acid, which are significantly enriched in YNUR, are flavor-active amino acids responsible for fresh sweet taste (Wang et al., 2023). According to Liu et al. (2025), it is speculated that abundant glycerophospholipid metabolites may competitively bind water during starch gelatinization, inhibiting excessive swelling of starch granules and amylose leaching, thereby modulating the texture of cooked rice.

In this study, conventional rice varieties with diverse genetic backgrounds were selected as control, which can effectively avoid the accidental deviation caused by single variety comparison. The quality and metabolic differences between YNUR and JXPR or HLJPR can still be stably distinguished under the multi-genotype control system, which shows that the results of this study have good stability and anti-interference ability.

### 4.3 Limitation analysis

There are still obvious limitations of experimental design in this study. First, there was an imbalance in genotypic matching among test materials. YNUR samples were a single cultivar, while JXPR and HLJPR samples were multi-variety germplasm groups. Genotypic difference is one of the critical factors contributing to the differentiation of sample quality. Furthermore, the rice samples were collected from three distinctly different ecological regions. Multiple environmental variations in altitude, latitude, climate, and soil physicochemical properties existed among these sampling sites, making it impossible to separate the independent effects of geographical ecological factors and cultivation patterns on rice quality. Therefore, the objective of this study is limited to comparing the quality differences between YNUR samples and JXPR and HLJPR samples, as well as exploring their correlations with metabolic profiles. Further experiments involving identical cultivars cultivated at different sites and single-variable water-controlled treatments will be conducted to further clarify the independent contributions and interactive mechanisms of genotype, ecological environment, and cultivation patterns to rice quality.

## 5 Conclusion

This study analyzed the quality characteristics of upland rice in Yunnan Plateau from many aspects. Compared with paddy rice, YNUR exhibited higher amylose content, lower eGI, higher gelatinization temperature, and superior thermal stability. The sensory advantages of YNUR are high elasticity, low viscosity, and high flavor perception intensity, but it has the disadvantages of high hardness and incomplete shape. YNUR contained a greater variety of volatile flavor substances, with relatively higher relative contents of fruity and baking aroma compounds. (Z)-2-nonenal, nonanal and 2-ethyl-3,5-dimethylpyrazine were the key differential odor components that distinguish upland-cultivated YNUR from flooded JXPR and HLJPR. Additionally, stress-responsive metabolites in YNUR were highly enriched, the amino acid synthesis pathway was the core regulatory pathway, and flavor amino acids were highly accumulated. These findings provide a theoretical basis for quality evaluation under water-saving upland rice cultivation and for utilizing drought-resistant germplasm. In this study, the limited number of sampling regions and rice varieties made it difficult to eliminate the potential influences of geographical environment and cultivation patterns on the analysis of rice quality.

## CRediT authorship contribution statement

**Ying Xiao:** Writing – original draft, Visualization, Methodology, Investigation, Formal analysis. **Zhenxing Wang:** Visualization, Methodology, Investigation. **Nandong Yue:** Writing – original draft, Investigation. **Yunfei Tuo:** Methodology. **Jiancheng Wen:** Methodology. **Liwei Guo:** Methodology. **Chunfang Zhao:** Methodology. **Xiahong He:** Supervision, Resources, Funding acquisition, Conceptualization. **Jinggui Nie:** Writing – review & editing, Validation, Supervision, Funding acquisition, Conceptualization.

## Ethical statement

The sensory evaluation protocol of this study has been approved by the Scientific Ethics Committee of Southwest Forestry University (Approval No.: SWFU-2025-0045). The experimental procedures were strictly performed in accordance with the relevant specifications of *Guidelines for Ethical and Professional Practice in Food Sensory Analysis*. All participants involved in the sensory evaluation voluntarily took part in the study on the basis of fully understanding the research content and potential risks, and have authorized the necessary use of their personal information.

## Ethics declaration

Written informed consent to take part in the study and to publish the article has been obtained from all participants or their legal representatives. The privacy rights of participants have been observed.

This study was performed in compliance with relevant laws, regulatory frameworks and guidelines where the research took place.

This study was conducted in accordance with Guidelines for Ethical and Professional Practice in Food Sensory Analysis.

This study was approved by the Scientific Ethics Committee of Southwest Forestry University.

(Approval No. SWFU-2025-0045).

## Declaration of competing interest

The authors declare that they have no known competing financial interests or personal relationships that could have appeared to influence the work reported in this paper.

## Data Availability

The data that has been used is confidential.
