# Supplementary material for: Quality divergence of Yunnan upland rice versus Jiangxi and Heilongjiang paddy rice revealed by physicochemical, sensory, volatile and metabolomic profiling
Source: Food Chem X. 2026 Sep 29;39:104539. doi: 10.1016/j.fochx.2026.104539 (PMC13656600; doi:10.1016/j.fochx.2026.104539)
Supplement: Supplementary file 1 [file mmc1.docx]

Fig. S1 Different sensory index scores (A) and total score (B) of rice samples. The results were expressed as mean ± SD.

Fig. S2 Correlation analysis between physicochemical indexes and the top 30 metabolites in Cluster 4 (A). Correlation analysis of taste indexs, sensory indexs and the top 30 metabolites in Cluster 4 (B). Correlation analysis between different odor compounds and the top 30 metabolites in Cluster 4 (C). Correlation analysis between physicochemical indicators and sensory qualities (D).

Table S1. Information of the 12 collected rice samples

| Rice varieties | Germplasm | Rice origin | | | Agronomic management | Abbr. |
| --- | --- | --- | --- | --- | --- | --- |
|  |  | Province | City | Location |  |  |
| Dianheyou 615 | Upland rice | Yunnan | Honghe | 23.37°N, 103.37°E | Upland rice and paddy rice experimental fields adopted uniform management practices for soil tillage, fertilization, weeding, and pest and disease control, differing only in water management. Compound fertilizer (N:P₂O₅:K₂O = 15:15:15) was applied as base fertilizer at 450 kg/hm². Urea was top-dressed at 120 kg/hm² during the tillering stage, and panicle fertilizer at 90 kg/hm² at the heading stage. Manual and chemical weeding was implemented uniformly throughout the whole growth period, and low-toxicity broad-spectrum pesticides were used for integrated pest and disease management.  Upland rice was cultivated under rainfed upland conditions with no continuous water layer; crop water supply relied on natural rainfall supplemented with manual irrigation at critical growth stages. Sufficient pre-sowing irrigation was applied before seeding. The soil was kept moist without standing water during seedling emergence and tillering stages, with slight supplementary irrigation in the absence of rainfall. At water-sensitive stages including jointing, booting, grain filling and seed setting, moderate irrigation was conducted under continuous drought conditions, while natural rainfall fully supported crop growth under normal precipitation.  Paddy rice was grown using conventional flooded cultivation with staged water management according to growth stages: a 1–2 cm shallow water layer was maintained from sowing to seedling emergence; a 3–4 cm shallow water layer was kept during early tillering, and moderate field drying was performed at late tillering to control tiller number; a 3–5 cm deep water layer was restored at jointing and booting stages; frequent shallow irrigation was applied during heading and flowering stages; alternate wetting and drying irrigation was adopted at grain filling and maturation stages, with water cut-off 7 d before harvest. | KY |
| Dianheyou 615 | Upland rice | Yunnan | Pu'er | 22.78°N, 100.97°E |  | LC |
| Dianheyou 615 | Upland rice | Yunnan | Nujiang | 25.82°N, 98.85°E |  | NJ |
| Dianheyou 615 | Upland rice | Yunnan | Qujing | 25.49°N, 103.79°E |  | TB |
| Shennong 508 | Paddy rice | Jiangxi | Yichun | 27.82°N, 114.42°E |  | SN |
| Dianheyou 615 | Paddy rice | Jiangxi | Ji'an | 27.09°N, 114.97°E |  | DHY |
| Huang Huazhan | Paddy rice | Jiangxi | Ganzhou | 25.83°N, 114.93°E |  | HHZ |
| 926 | Paddy rice | Jiangxi | Jiujiang | 29.66°N, 115.95°E |  | 926/J926 |
| Rice Blossom No.2 | Paddy rice | Heilongjiang | Harbin | 45.80°N, 126.53°E |  | DHX |
| Sanjiang 6 | Paddy rice | Heilongjiang | Jiamusi | 46.82°N, 130.37°E |  | JSJ |
| Mudrang 1 | Paddy rice | Heilongjiang | Suihua | 46.64°N, 126.99°E |  | XS |
| Sui-Tsai 18 | Paddy rice | Heilongjiang | Jixi | 45.30°N, 130.98°E |  | ZBD |

Table S2. Sensory Scoring Scale

| Evaluation Indicator | Total Score | Corresponding Score | Description of Scoring Criteria |
| --- | --- | --- | --- |
| Rice grain integrity | 5 | 5 | Intact rice grains with uniform shape; almost no broken grains or chips, plump and neat in appearance |
|  |  | 3 | Mostly intact grains with a small amount of fractures or broken rice; overall shape is acceptable |
|  |  | 1 | Severe grain breakage with a high proportion of broken rice; incomplete grain morphology |
| Cold rice texture | 5 | 5 | Rice grains maintain good elasticity after cooling, without hardness or agglomeration |
|  |  | 3 | Slightly hard grains after cooling with mild agglomeration |
|  |  | 1 | Hard grains with severe agglomeration after cooling, rough and dry texture |
| Color | 8 | 8 | Uniform milky white or pearlescent white grains, free of yellowing, discoloration and chalkiness |
|  |  | 5 | Basically uniform grain color with slight dullness or minor color difference; no obvious yellowing |
|  |  | 2 | Uneven grain color, yellow and dull, with prominent discoloration or chalkiness |
| Gloss | 8 | 8 | Smooth and glossy grain surface with even natural luster and translucent texture |
|  |  | 5 | Slight surface gloss with moderate brightness, no obvious dryness |
|  |  | 2 | Dull and matte grain surface, dry and coarse without luster |
| Softness | 8 | 8 | Moderate hardness; soft but not mushy on the palate with pleasant chewing sensation |
|  |  | 5 | Slightly too hard or too soft grains with mediocre chewing experience |
|  |  | 2 | Excessively hard or mushy grains with poor mouthfeel and difficult chewing |
| Flexibility | 8 | 8 | Highly elastic grains with distinct springback during chewing and good toughness |
|  |  | 5 | Moderate elasticity; slight toughness with unnoticeable springback when chewed |
|  |  | 2 | No elasticity; mushy or hard grains without springback, loose or rigid mouthfeel |
| Adhesion | 8 | 8 | Moderate cohesiveness between grains; cohesive clumps without sticking to teeth |
|  |  | 5 | Average viscosity, slightly sticky to teeth or relatively loose |
|  |  | 2 | Excessively sticky clumps or fully loose grains, strongly adhering to teeth |
| Odor | 25 | 25 | Distinct fresh rice aroma, natural, pure, rich and pleasant; free of off-flavors, stale odor or moldy smell |
|  |  | 15 | Faint rice aroma with basically no off-odors; mediocre overall odor and inconspicuous characteristic fragrance |
|  |  | 5 | Loss of fresh rice scent with prominent stale, moldy, rancid or other undesirable off-flavors |
| Taste | 25 | 25 | Sweet and fresh taste, rich rice flavor with long aftertaste, harmonious and pure taste without off-flavors or bitterness |
|  |  | 15 | Bland taste with subtle rice aroma; no obvious bad taste and ordinary flavor performance |
|  |  | 5 | Poor mouthfeel accompanied by off-flavors, bitterness, astringency or tastelessness; faint rice fragrance and obvious flavor deterioration |

Table S3. Odor description, threshold, and relative odor activity value (ROAV) of odor compounds with Discrimination power > 0.800 and peak area > 100

| CAS | Compound | Odor description | Odor threshold | ROAV | | | | | | | | | | | |
| --- | --- | --- | --- | --- | --- | --- | --- | --- | --- | --- | --- | --- | --- | --- | --- |
|  |  |  |  | KY | LC | NJ | TB | SN | DHY | HHZ | 926 | DHX | JSJ | XS | ZBD |
| 75-07-0 | Acetaldehyde | Fruit | 0.09 | 0.00 | 0.00 | 0.00 | 0.00 | 0.00 | 0.00 | 0.00 | 0.00 | 0.00 | 0.00 | 0.00 | 0.00 |
| 66-25-1 | Hexanal | Fruit | 0.04 | 0.08 | 0.09 | 0.31 | 0.10 | 0.12 | 0.06 | 0.10 | 0.03 | 0.05 | 0.07 | 0.12 | 0.10 |
| 124-19-6 | N-nonanal | Fruit | 0.00034 | 2.17 | 2.42 | 9.57 | 3.57 | 5.20 | 1.79 | 4.76 | 0.70 | 2.23 | 2.25 | 5.20 | 5.29 |
| 60784-31-8 | (Z)-2-nonenal | Herb | 0.00008 | 2.21 | 2.98 | 17.86 | 5.81 | 4.55 | 1.47 | 7.93 | 0.99 | 3.65 | 4.00 | 8.24 | 13.07 |
| 600-14-6 | 2,3-Pentanedione | Baking | 0.02 | 0.03 | 0.04 | 0.11 | 0.03 | 0.05 | 0.02 | 0.06 | 0.01 | 0.03 | 0.02 | 0.03 | 0.03 |
| 470-82-6 | 1,8-Cineole | Herb | 0.06 | 0.00 | 0.00 | 0.00 | 0.00 | 0.00 | 0.00 | 0.01 | 0.00 | 0.00 | 0.00 | 0.00 | 0.00 |
| 99-85-4 | Gamma-terpinene | Herb | 55 | 0.00 | 0.00 | 0.00 | 0.00 | 0.00 | 0.00 | 0.00 | 0.00 | 0.00 | 0.00 | 0.00 | 0.00 |
| 607-91-0 | Myristicin | Fat | 0.0015 | 0.07 | 0.10 | 0.45 | 0.12 | 0.12 | 0.10 | 0.41 | 0.06 | 0.06 | 0.12 | 0.09 | 0.28 |
| 106-42-3 | 1,4-Dimethylbenzene | Wood | 0.8 | 0.00 | 0.00 | 0.00 | 0.00 | 0.00 | 0.00 | 0.00 | 0.00 | 0.00 | 0.00 | 0.00 | 0.00 |
| 13643-08-8 | 2,4-Octadiene | Fat | 12 | 0.00 | 0.00 | 0.00 | 0.00 | 0.00 | 0.00 | 0.00 | 0.00 | 0.00 | 0.00 | 0.00 | 0.00 |
| 629-59-4 | Tetradecane | Fruit | 5 | 0.00 | 0.00 | 0.00 | 0.00 | 0.00 | 0.00 | 0.00 | 0.00 | 0.00 | 0.00 | 0.00 | 0.00 |
| 28588-74-1 | 2-Methyl-3-furanthiol | Baking | 0.0000025 | 100.00 | 100.00 | 100.00 | 100.00 | 100.00 | 100.00 | 100.00 | 100.00 | 100.00 | 100.00 | 100.00 | 76.98 |
| 4466-24-4 | 2-Butylfuran | Fruit | 10 | 0.00 | 0.00 | 0.00 | 0.00 | 0.00 | 0.00 | 0.00 | 0.00 | 0.00 | 0.00 | 0.00 | 0.00 |
| 13925-03-6 | 2-Ethyl-6-methylpyrazine | Baking | 0.04 | 0.01 | 0.01 | 0.04 | 0.01 | 0.01 | 0.03 | 0.08 | 0.02 | 0.01 | 0.01 | 0.01 | 0.05 |
| 13925-07-0 | 2-Ethyl-3,5-dimethylpyrazine | Baking | 0.0000105 | 4.57 | 3.69 | 64.70 | 8.34 | 5.92 | 27.07 | 34.40 | 33.46 | 5.20 | 5.91 | 4.39 | 100.00 |
